# Clinical course and outcome of essential thrombocythemia and prefibrotic myelofibrosis according to the revised WHO 2016 diagnostic criteria

**DOI:** 10.18632/oncotarget.21594

**Published:** 2017-10-06

**Authors:** Elisa Rumi, Emanuela Boveri, Marta Bellini, Daniela Pietra, Virginia V. Ferretti, Emanuela Sant’Antonio, Chiara Cavalloni, Ilaria C. Casetti, Elisa Roncoroni, Michele Ciboddo, Pietro Benvenuti, Benedetta Landini, Elena Fugazza, Daniela Troletti, Cesare Astori, Mario Cazzola, the Associazione Italiana per la Ricerca sul Cancro Gruppo Italiano Malattie Mieloproliferative Investigators

**Affiliations:** ^1^ Department of Molecular Medicine, University of Pavia, Pavia, Italy; ^2^ Department of Hematology Oncology, Fondazione Istituto di Ricovero e Cura a Carattere Scientifico (IRCCS) Policlinico San Matteo, Pavia, Italy; ^3^ Anatomic Pathology Section, Fondazione Istituto di Ricovero e Cura a Carattere Scientifico (IRCCS) Policlinico San Matteo, Pavia, Italy; ^4^ Department of Oncology, Division of Hematology, Ospedale San Luca, Lucca, Italy

**Keywords:** myelofibrosis, thrombocythemia, prefibrotic, WHO, diagnostic criteria

## Abstract

The recently revised World Health Organization (WHO) classification of myeloid neoplasms recognizes prefibrotic myelofibrosis (prePMF) as a distinct entity, characterized by well-defined histopathologic features together with minor clinical criteria (leukocytes, anemia, increased LDH, splenomegaly). The aim of the study was to examine the clinical relevance of distinguishing prePMF from essential thrombocythemia (ET). We identified in our database all patients affected with ET, prePMF and primary myelofibrosis (PMF) diagnosed according to 2008 WHO criteria with a bone marrow fibrosis grade 0-1 at diagnosis and one DNA sample to define the mutational status. The bone marrow morphology of all 404 identified patients was reviewed by an expert pathologist and patients were reclassified according to the 2016 WHO criteria. After reclassification, our cohort included 269 ET, 109 prePMF, and 26 myeloproliferative neoplasm unclassificable. In comparison with ET, patients with prePMF had higher leukocyte count, lower hemoglobin level, higher platelet count, higher LDH values, and higher number of circulating CD34-positive cells; they showed more frequently splenomegaly (all *P* values < ·001). *CALR* mutations were more frequent in prePMF than in ET (35·8% vs 17·8%, *P* < ·001). PrePMF patients had shorter overall survival (*P* < ·001) and a trend to a higher incidence of leukemic evolution (*P* ·067) compared to ET patients, while they did not differ in terms of thrombotic and bleeding complications. In conclusion, ET and prePMF diagnosed according to 2016 WHO criteria are two entities with a different clinical phenotype at diagnosis and a different clinical outcome.

## INTRODUCTION

The recently revised World Health Organization (WHO) classification of myeloid neoplasms for the first time recognized prefibrotic PMF as a distinct entity, characterized by well-defined histopathologic features together with minor clinical criteria (anemia, leukocytosis >= 11 x10^9^/L, palpable splenomegaly, increased LDH) [1]; however, the diagnostic profiles of essential thrombocythemia (ET) (traditionally defined by isolated thrombocytosis in the absence of relevant bone marrow fibrosis and megakaryocyte atypia) and primary myelofibrosis (PMF) (characterized by extramedullary hematopoiesis, bone marrow reticulin or collagen fibrosis, increased granulocytopoiesis, megakaryocyte hyperplasia and dysplasia) have been challenged since 2001, when the WHO classification recognized, within the category of PMF, a novel group of patients, namely early/prefibrotic MF (prePMF), which often presented marked thrombocytosis in the peripheral blood, granulocytic and atypical megakaryocytic proliferation with minimal, if any, fibrosis in the bone marrow [2].

During the last decade, the accurate histologic distinction between “true” ET and prePMF according to the WHO criteria has been questioned due to a substantial inter-observer variability [3]; though much debated, this distinction has been reported to have deep prognostic implications. In the largest series of ET and prePMF cases reported to date, with both local and central review by expert hemopathologists, the outcome of prePMF patients was significantly worse in terms of overall survival (89% vs 76% at 10 years; 80% vs 59% at 15 years) and rates of evolution to acute leukemia (5·8% vs 0·7% at 10 years; 11·7% vs 2·1% at 15 years) and to overt myelofibrosis (12·3% vs 0·8% at 10 years; 16·9% vs 9·3% at 15 years), even though event-free survival was still relatively long in prePMF patients and thrombosis rates were comparable [4].

On the other hand, major bleeding rate during follow up was almost double in prePMF patients, compared to ET ones (1.39% vs 0.79% patients per year, respectively); while extreme thrombocytosis was not a risk factor for bleeding in ET and prePMF patients which were not treated with low-dose aspirin, the use of this antiplatelet agent seemed to increase the bleeding risk in prePMF and seemed to have a synergistic hemorrhagic effect in ET patients with extreme thrombocytosis [5].

These observations further underline how an accurate distinction between ET and prePMF could have prognostic and therapeutic implications. Similarly, a recent paper demonstrated that prefibrotic and overt PMF diagnosed according to 2016 WHO criteria show different patterns of presentation, survival and intrinsic disease progression [6].

In the current study, we compared two cohorts of 2016 WHO-defined ET and prePMF patients in terms of clinical features at diagnosis and outcome. To accomplish this aim, we identified a cohort of patients previously diagnosed (2008 WHO criteria) as ET, prePMF or PMF (with bone marrow fibrosis grade 0 or 1), followed at our institution; bone marrow morphology was, then, reviewed and diagnosis was redefined according to the revised WHO criteria.

## RESULTS

### Presenting hematologic and clinical features of ET, prePMF and MPNu diagnosed according to the new 2016 WHO criteria

After histological revision and clinical reclassification according to the new 2016 WHO criteria, our cohort included 269 patients with ET, 109 patients with prePMF and 26 patients with MPNu.

**Figure 1:**
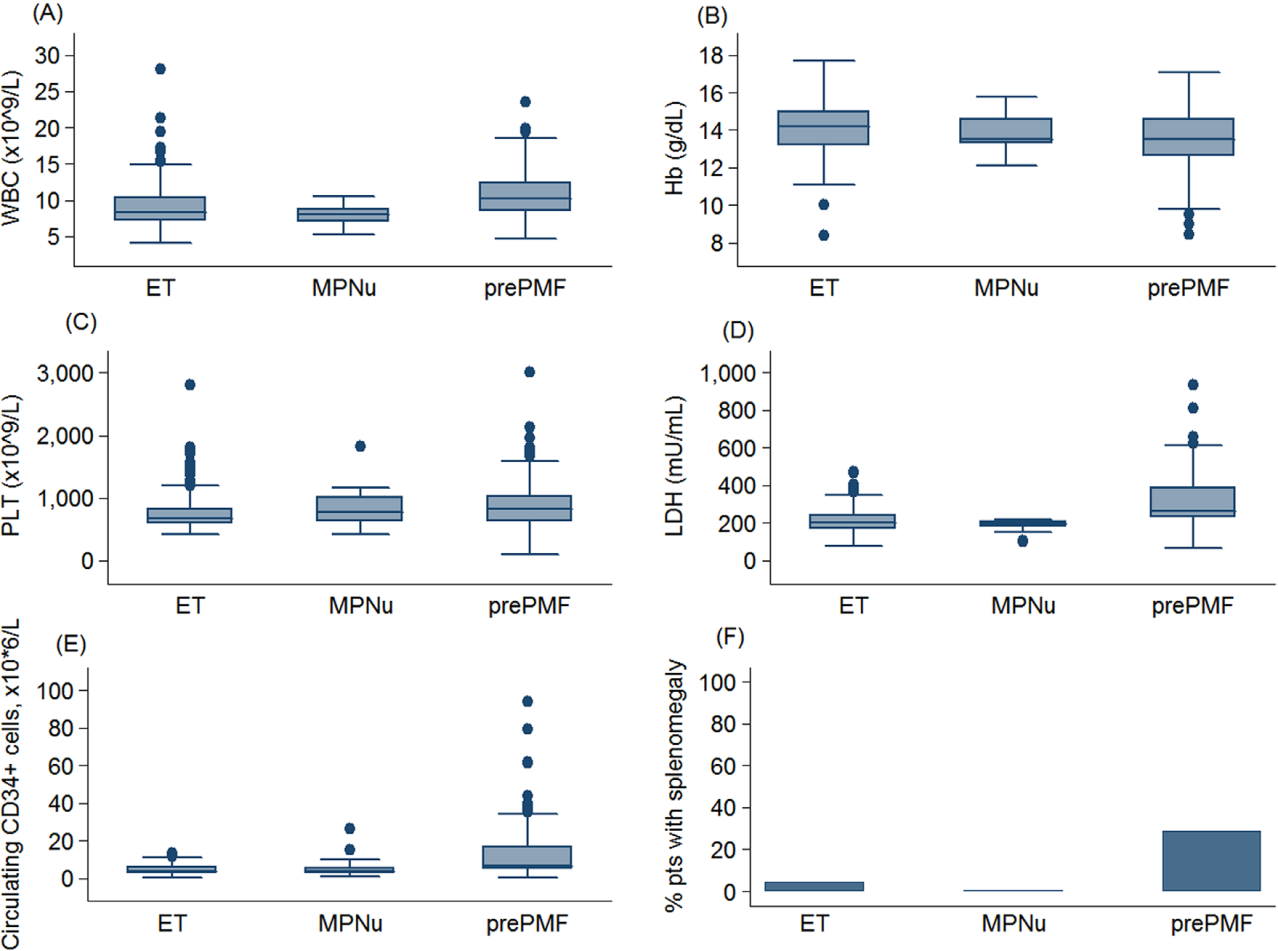
Main hematologic parameters in patients with essential thrombocythemia and prefibrotic myelofibrosis diagnosed according to the new 2016 WHO criteria Data are shown in a box plot depicting the upper and lower adjacent values (highest and lowest horizontal line, respectively), upper and lower quartile with median value (box), and outside values (dots). The figure shows **(A)** leukocyte count (WBC), **(B)** hemoglobin (Hb), **(C)** platelet count (PLT), **(D)** lactate dehydrogenase level (LDH), **(E)** circulating CD34-positive cells, **(F)** the percentage of patients with splenomegaly.

Table 1 reports demographic, clinical characteristics and hematological parameters at diagnosis of the patients studied according to their new 2016 WHO diagnosis. Compared to patients with ET, patients with prePMF had higher leukocyte count, lower hemoglobin levels, higher platelet count, higher LDH values, higher number of circulating CD34-positive cells, and showed more frequently splenomegaly (*P* values in Table 1). Extreme thrombocytosis (PLT count > 1000 × 10^9^/L) was more frequent in patients affected with prePMF than in patients affected with ET (31·2% vs 11·5%, *P* <·001). The main hematological and clinical differences are shown in Figure 1. ET and MPNu did not differ in terms of leukocyte count, hemoglobin, platelet count, LDH, circulating CD34-positive cells and splenomegaly.

**Table 1:** Clinical features of patients according to the new 2016 WHO criteria

N°	ET (A)	MPNu (B)	prePMF (C)	*P*		
	269	26	109	A vs B	B vs C	A vs C
Sex (male/female)	105/164(39%/61%)	7/19(27%/73%)	55/54(51%/49%)	·291	·047	·051
Age at onset, years, median (range)	53·1(17·4-88·5)	44·3(18·2-79·4)	54·7(15·6-83)	·015	·019	·938
Hemoglobin, g/dL, median (range)	14·2(8·4-17·7)	13·5(12·1-15·8)	13·5(8·5-17·1)	·101	·390	< ·001
WBC count, x 10^9^/L, median (range)	8·3(4·2-28)	8·1(5·3-10·6)	10·3(4·7-23·5)	·117	< ·001	< ·001
PLT count, x 10^9^/L, median (range)	677(450-2810)	765(414-1825)	823(97·8-3000)	·057	·643	< ·001
Splenomegaly, no. (%)	12 (4·5%)	0 (0%)	31 (29%)	·609	·001	< ·001
LDH, mU/mL, median(range)	200(77-472)	194(100-220)	265(66-935)	·133	< ·001	< ·001
Circulating CD34+ cells, x 10^6^/L, median(range)	3·6(0·4-13·2)	3·6(0·6-26·5)	6·6(0·2-94·1)	> ·900	·001	< ·001
Mutational status, no. (%)				·419	·774	<·001
*JAK2* V617F	179 (66·5%)	17 (65·3%)	57 (52·3%)			
*CALR*	48 (17·8%)	7 (26·9%)	39 (35·8%)			
*MPL*	9 (3·4%)	1 (3·9%)	7 (6·4%)			
Triple neg	33 (12·3%)	1 (3·9%)	6 (5·5%)			
Subtypes of *CALR* mutations no. (%)				0·242	0·220	0·081
Type 1-like	23 (47·9%)	4 (57·1%)	27 (69·2%)			
Type 2-like	24 (50%)	2 (28·6%)	12 (30·8%)			
Other	1 (2·1%)	1 (14·3%)	0 (0%)			

### Mutational status of essential thrombocythemia and prefibrotic myelofibrosis diagnosed according to 2016 WHO

Of the 269 patients with ET, 179 (66·5%) carried *JAK2* V617F, 48 (17·8%) a *CALR* exon 9 indel, 9 (3·4%) an *MPL* mutation, and 33 (12·3%) had nonmutated *JAK2*, *CALR*, and *MPL* (ie, triple-negative patients). Of the 109 patients with prePMF 57 (52·3%) carried *JAK2* V617F, 39 (35·8%) a *CALR* exon 9 indel, 7 (6·4%) an *MPL* mutation, and 6 (5·5%) were triple-negative, as reported in Table 1. The higher frequency of *CALR* mutations in prePMF compared to ET (35·8% vs 17·8%, *P* <·001) might contribute to the high level of platelet count observed in prePMF and to the higher frequency of extreme thrombocytosis (> 1000 × 10^9^/L) observed in prePMF.

We estimated the *JAK2* mutant allele burden at diagnosis in 152 out of 179 patients with ET and in 43 out of 57 patients with prePMF. The median allele burden was 14·7% (range 1 to 65·5%) in patients with ET at clinical onset versus 29·9% (range 8·1 to 93·1) in those with prePMF (*P* <·001).

Then we considered the different types of *CALR* exon 9 mutations, classified as type 1-like and type-2 like according to our previous study [7]. The frequency of type-1 like mutations in patients with prePMF seems higher than that observed in patients with ET (69·2% vs 47·9%), but the difference is not statistically significant (*P* = ·081), as reported in Table 1.

### Clinical course of essential thrombocythemia and prefibrotic myelofibrosis diagnosed according to 2016 WHO

The median follow-up of the study population was 5·4 years (range 0-30·2 years).

**Figure 2:**
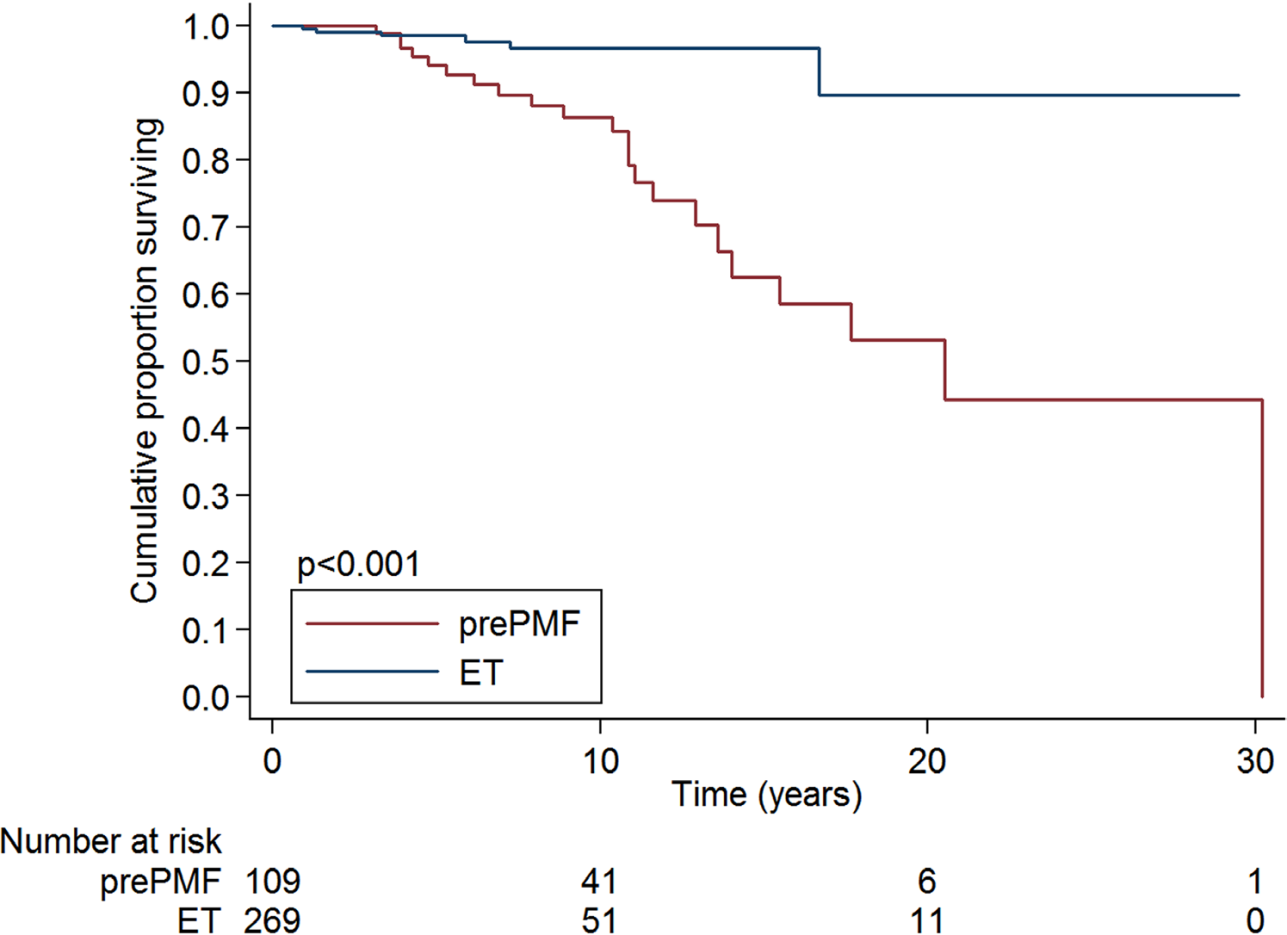
Overall survival of patients with essential thrombocythemia and prefibrotic myelofibrosis diagnosed according to the new 2016 WHO criteria ET patients had a better overall survival than prePMF patients (overall survival at 10-years 96·6% vs 86·4%, *P* <·001).

Overall survival at 10-years was 86·4% in prePMF patients and 96·6% in ET patients, as shown in Figure 2. In univariate analysis, ET patients had a better overall survival than prePMF patients (HR 0·18, 95%CI: 0·07-0·45, *P* <·001). In a multivariate analysis corrected for age, ET patients maintained a better overall survival compared to prePMF patients (HR 0·17, 95%CI: 0·07-0·42, *P* <·001). The overall survival was not influenced by the mutational status, both in ET (*P* = ·343) and in prePMF (*P* = ·382).

**Figure 3:**
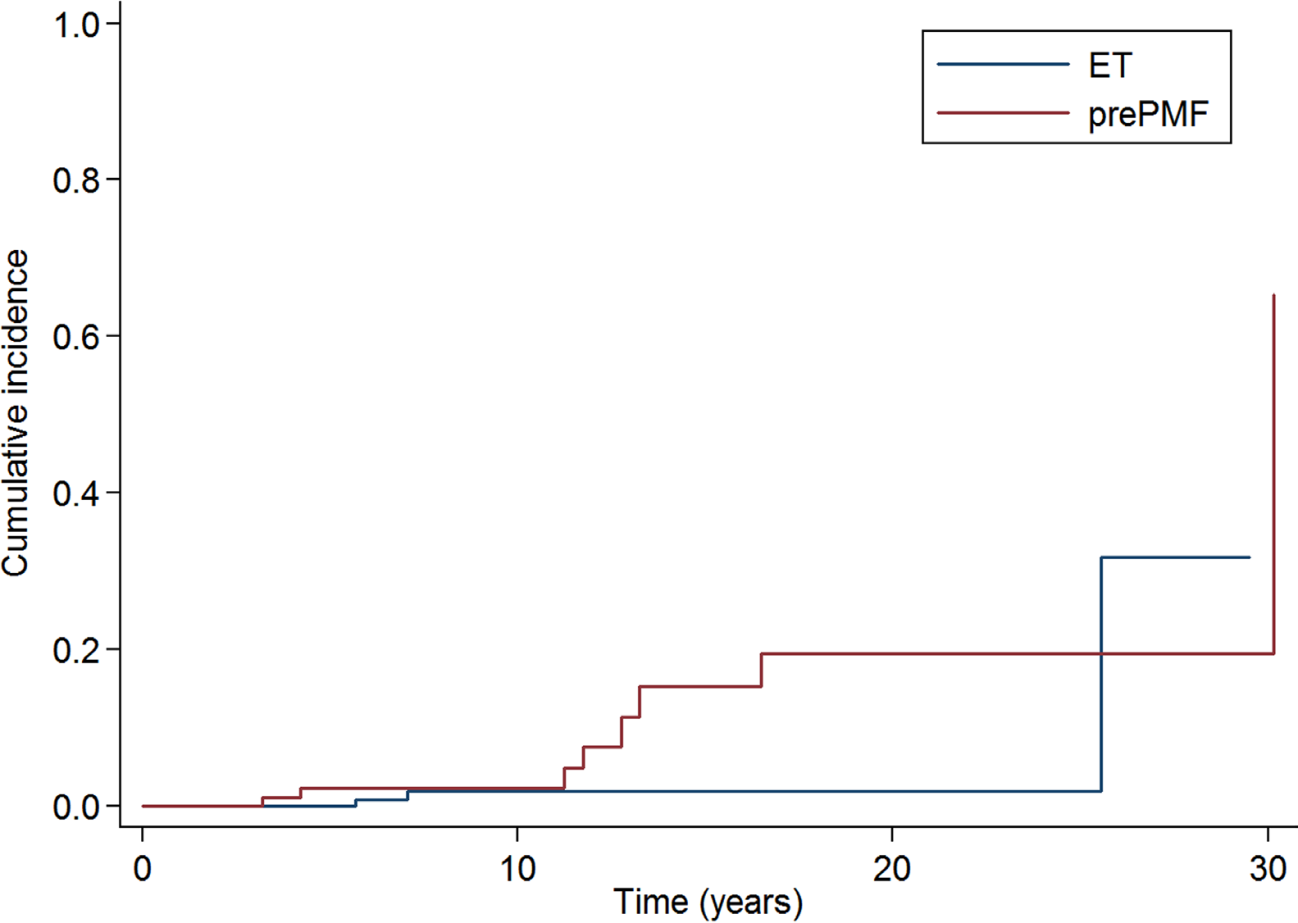
Cumulative incidence of leukemic evolution in patients with essential thrombocythemia and prefibrotic myelofibrosis diagnosed according to the new 2016 WHO criteria The 10-years cumulative incidence of leukemia was 2·3% (95% CI: 0·4-7·3%) in prePMF and 1·9% (95% CI: 0·4-6%) in ET, with a trend (*P* ·067) to a higher risk of leukemic evolution in prePMF.

The 10-years cumulative incidence of leukemia was 2·3% (95%CI: 0·4-7·3%) in prePMF and 1·9% (95%CI: 0·4-6%) in ET, with a trend (*P* ·067) to a higher risk of leukemic evolution in prePMF (Figure 3). To evaluate whether the incidence of leukemic evolution might be influenced by the higher frequency of *CALR* mutations in prePMF compared to ET, we performed a multivariate analysis including diagnosis (ET/prePMF) and *CALR* mutation (mutated/not mutated) but we did not observe an impact of *CALR* mutation (*P* =·387).

The 10-years cumulative incidence of thrombosis was 18.5% (95%CI: 10·7-27·8%) in prePMF and 18% (95%CI: 11·7-25·4%) in ET, without any significant difference between the two diagnosis (*P* >·90).

The 10-years cumulative incidence of bleeding was 14·9% (95%CI: 8·2-23·6%) in prePMF and 19·6% (95%CI: 13·2-27%) in ET, without any significant difference between the two diagnosis (*P* =·503). The incidence of bleeding was not different between the two diagnosis also when restricting the analysis to the subgroup of 112 patients with a low risk disease (95 ET patients and 17 prePMF patients) who did not receive cytoreduction (*P* =·751).

**Figure 4:**
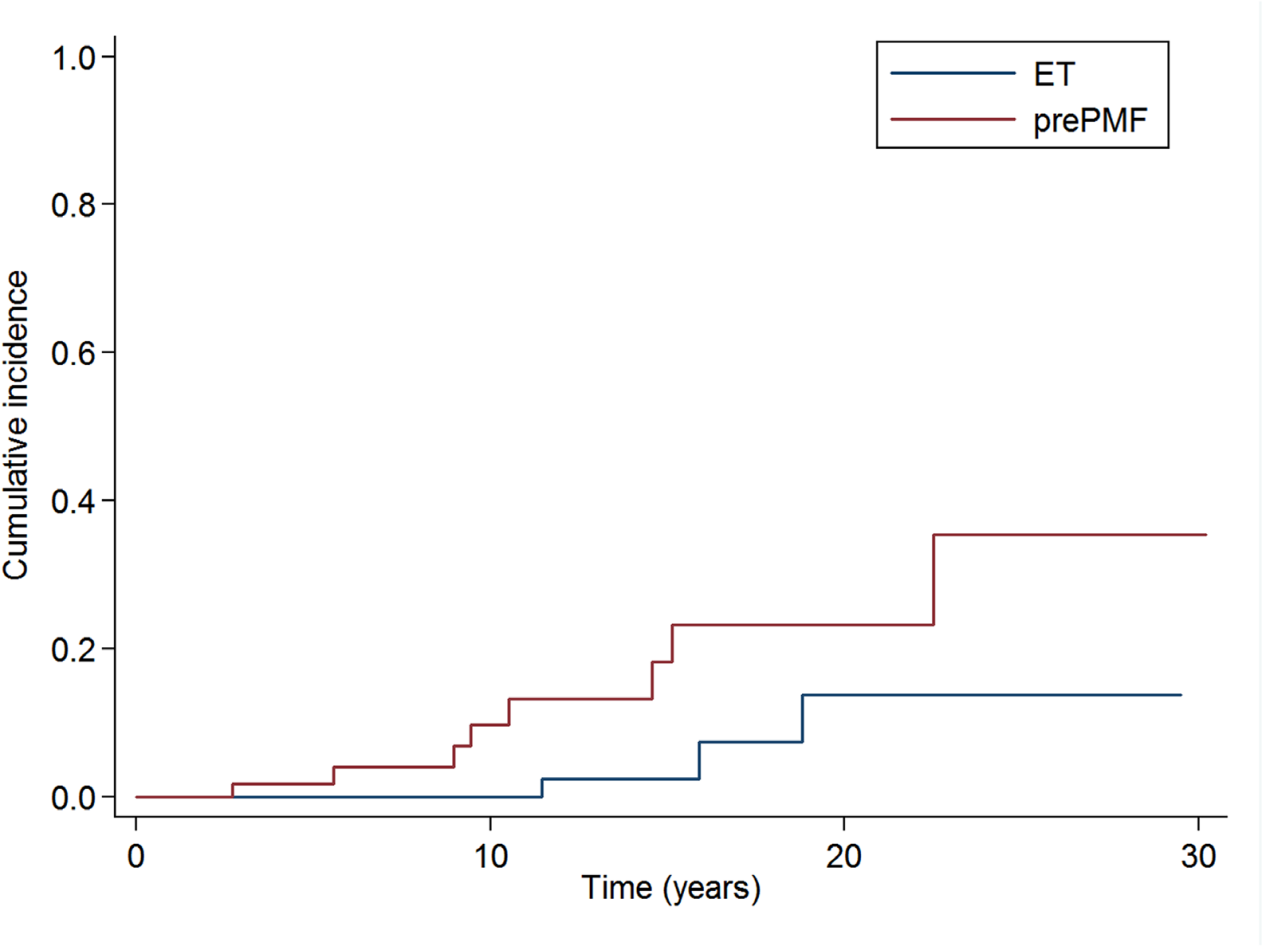
Cumulative incidence of myelofibrotic evolution in the subgroups of 358 patients affected with “old” ET reclassified according to the new 2016 WHO criteria The cumulative incidence of overt myelofibrosis at 10 years was significantly higher in the “old” ET reclassified as prePMF than in the “old” ET reclassified as ET (9·7% vs 0%, *P* ·033).

Finally, we analyzed the subgroup of “old” ET diagnosed according to 2008 WHO criteria. Of 358 “old” ET, 268 were reclassified as ET, 25 as MPNu and 65 as prePMF. The “old” ET reclassified as prePMF had a higher risk of overt myelofibrotic evolution compared to the “old” ET reclassified as ET (cumulative incidence of overt myelofibrosis at 10 years 9·7% vs 0%, *P* ·033), as reported in Figure 4.

## DISCUSSION

In 1976 a group of European pathologists described for the very first time a subtype of chronic myeloproliferative disorder that was characterized by megakaryocytic and granulocytic hyperplasia, with atypical megakaryocyte morphology, but without any significant bone marrow fibrosis [8]. It was first conceived as a variant of Chronic Myeloid Leukemia, but was later recognized as an early, hyperplastic stage of PMF, following extensive studies by the Cologne group of pathologists, that were mainly focused on bone marrow histomorphology of patients with thrombocytosis due to an underlying chronic myeloproliferative disorder [9].

It has been formally introduced in the WHO classification of tumours in 2001 [2], confirmed and revised in 2008 [10], and then defined as a distinct clinico-pathologic entity in the recent 2016 revision, as prefibrotic PMF (prePMF) [1]. Histological hallmarks of prePMF, as reported by the WHO classification, include bone marrow hypercellularity, with prominent granulocytic and megakaryocytic hyperplasia, often associated with a reduction of red blood cell precursors, without (or with only minor) reticulin fibrosis (MF-0 or MF-1); megakaryocytes abnormalities include translocation towards the endosteal space, dense clustering, a high variability in size, ranging from small to giant elements, with atypical maturation (nuclear hypolobulation, irregular nuclear foldings, with a bulbous or cloud-like appearance, elevated nuclear-cytoplasmic ratio and increased frequency of bare nuclei). These histological features, together with the recently introduced clinical criteria [1], allow clinicians and pathologists to distinguish prePMF cases and ET ones, that are traditionally defined by an age-matched bone marrow cellularity with a predominant megakaryopoiesis, composed of large to giant cell forms, with extensive nuclear folding (hyperlobulated nuclei), mature-appearing cytoplasm, randomly distributed or in very loose clusters within the bone marrow space, without significant increase in reticulin, nor erythroid o granulocytic proliferation.

Even though it has been formally recognized by the WHO classification and has been widely accepted as a separate entity among the Philadelphia-negative MPN, the diagnostic process of prePMF cases is still largely debated, mainly due to concerns regarding the reliability and reproducibility of the histopathological criteria, especially those regarding megakaryocytes morphology, size and clustering [3, 11–13].

Major criticisms regarded the lack of precise guidance on the relative ‘weight’ of different morphological features and the qualitative nature of the criteria defined by the WHO (as opposed to a quantitative assessment, that has been proposed as a more accurate and standardized procedural approach) [14]; to complicate things further, it's not so uncommon for a bone marrow sample to show some morphological elements consistent with true ET, together with features that are thought to reflect prePMF or even overt PMF. However, several studies showed that, among well trained hemopathologists, WHO criteria proved to be reliable and consistently applicable, so that prePMF and ET cases could be adequately identified [4, 15, 16]. In this regard, it's worth noting that in one of these studies [4], which involved 1104 patients previously diagnosed and treated as ET in seven international centers of excellence in the field of myeloproliferative neoplasms, a not negligible percentage of ET diagnosis (180 patients, 16%) were revised as prePMF after central, outcome-blinded bone marrow review by J Thiele, one of the Authors of the WHO chapters of MPN diagnostic criteria.

Irrespective of the controversies regarding the role of morphology in distinguishing between these two entities, the clinical relevance of an accurate diagnosis has been already consistently reported [4, 5, 17, 18], and the differences in terms of clinical features, natural history and outcome prediction between ET and prePMF, together with the consequent translational implications, have been extensively reviewed [19]. Indeed, the above mentioned studies showed that prePMF patients (irrespective of their age) had higher leukocyte and platelet count, lower hemoglobin value, higher serum LDH, higher circulating CD34+ cells and higher frequency of palpable splenomegaly, when compared to ET ones; survival rates, leukemic transformation and rates of progression to overt myelofibrosis were different as well, being significantly worse for prePMF patients, thus underlining that a careful and proper diagnosis bears prognostically relevant information.

The aim of the current study was to validate these observations following the recent revision of the WHO classification [1], that introduced a set of unique clinical criteria (different from those needed to establish a diagnosis of PMF) to define prePMF cases in a more stringent and rigorous way, thus complementing and enhancing the diagnostic role of histopathology. Minor clinical criteria, namely anemia not attributed to a comorbid condition, leukocytosis ≥11 × 10^9/L, palpable splenomegaly and LDH value above the upper limit of normal of institutional reference range, were previously identified as potential diagnostic and prognostic features [19, 20].

All our patients satisfied the 2016 WHO criteria, in contrast with the recent paper published by Jeryczynski, in which 8.8% of pre-PMF patients failed to show one of the clinical criteria required for pre-PMF diagnosis [21]. Our results confirm previous observations regarding significant differences in terms of clinical phenotype between prePMF and ET patients [4, 17, 18], the former presenting with higher leukocyte and platelet counts, lower hemoglobin values, higher LDH and circulating CD34-positive cells, and higher incidence of palpable splenomegaly. On the other hand, MPNu and ET cases were not significantly different in terms of blood counts, laboratory data and clinical findings, thus supporting the enhanced diagnostic power of combining clinical and morphological findings to define a clear-cut prePMF diagnosis. According to this observation and in contrast with the recent austrian paper [21], we reinforce our opinion regarding the identification of the MPNu category, including patients who present with thrombocytosis, have a bone marrow biopsy suggestive of prePMF but lack any of the minor criteria [22, 23].

To the best of our knowledge, no study has reported a significant difference in driver mutations frequency in prePMF and ET cases, while in our cohort we observed a higher incidence of *CALR* mutations among prePMF cases, compared to ET, that might explain, at least in part, the differences in platelet counts and in the occurrence of extreme thrombocytosis. Considering the *JAK2* V617F mutant burden we observed a higher burden in prePMF than in ET, thus confirming a previous observation by Hussein et al [24].

In terms of outcome, our study confirms that prePMF and ET patients share the same thrombotic risk, which can be often underestimated; our data together with previously reported observations [4, 17, 18], suggest that prePMF patients should be thoroughly informed, cardiovascular risk factors should be adequately managed and attention should be given not only to platelet and leukocyte counts, but to hematocrit level as well, since at least a fraction of prePMF patients presents with erythrocytosis (in the cohort reported by Barosi et al. 7·6% of female prePMF patients had hemoglobin values higher than 16·5 g/dl, which were consistent with a PV diagnosis according to WHO 2008) [18].

At the same time, prophylaxis with low-dose aspirin is still much debated, in particular for otherwise low-risk patients with extreme (higher than 1000×10^9/L) thrombocytosis, since it has been shown to have a synergistic hemorrhagic effect, particularly in (but no limited to) prePMF patients [5]. In this regard, however, our study did not show a significant difference in terms of major bleeding incidence, although the occurrence of extreme thrombocytosis was more frequent in prePMF. The incidence of bleeding did not differ also when considering only the subgroup of patients at low risk not receiving cytoreduction.

Finally, our analysis supports the hypothesis that WHO-defined prePMF and ET cases have different natural histories, with the former displaying a significantly lower overall survival, a higher risk of evolution to overt myelofibrosis and a trend towards a higher risk of leukemic evolution, when compared to ET patients. We did not observe an impact of mutational status on the overall survival in prePMF, in contrast with the recent paper by Jeryczynksi et al. who observed a negative impact of the *JAK2* mutation in pre-PMF patients in comparison with *CALR* positive patients [21]. It should be noted that we evaluated the molecular status in the whole cohort of patients while they evaluated only 50% of patients for whom the mutational status was available. This might explain the different results.

Notably, we observe a lower risk of myelofibrotic evolution among the subgroup of ‘old’ ET, reclassified as ET after histological review (10 years cumulative incidence of overt myelofibrosis 0% vs 9·7% for those cases that were initially diagnosed as ET and then reclassified as prePMF). The rate of progression to overt PMF in prePMF is in line with previously published results [4, 19] and in striking contrast with the unexpected high rate (36.9% at 10 years) observed in the recent austrian paper [21]. There was only a trend towards a higher risk of leukemic evolution in prePMF, but multivariate analysis demonstrated that this is not attributable to the “hypothetical benign” effect of higher frequency of *CALR* mutations in prePMF.

Taken together, our results support the value of performing a bone marrow biopsy at disease onset – in particular in younger patients presenting with an isolated thrombocytosis – and the relevance of a strict adherence to the 2016 WHO-defined criteria, combining morphological and clinical data, for an accurate diagnosis of MPN subtype, since it has consistently been shown to enhance our prognostic ability and to add valuable information that can have translational consequences on our management strategies. Further studies are needed to integrate the molecular data with histopathology and clinical phenotype in order to develop a future molecular classification.

## MATERIALS AND METHODS

### Study population and definitions

This study was approved by the local institutional Ethics Committee. The procedures followed were in accordance with the Helsinki Declaration of 1975, as revised in 2000, and samples were obtained after patients had provided written informed consent.

Criteria to be included in the following study were a diagnosis of ET or PMF according to 2008 WHO criteria [10] and a) a bone marrow biopsy at disease onset with fibrosis grade 0 or 1 and b) one DNA sample available for molecular studies. Paraffin sections were stained with Gomori's silver impregnation technique and fibrosis was assessed semi-quantitatively following the European consensus guidelines [25].

Of 2900 patients affected with MPN diagnosed at the Department of Hematology Oncology, Fondazione IRCCS Policlinico S. Matteo Pavia, Italy, between 1983 and 2015, 404 cases with ET (n=358) and PMF (n=46) satisfied the above criteria and have been included in the present work. The bone marrow morphology of all 404 identified patients was reviewed by an expert pathologist. Then, we reclassified patients according to the new 2016 WHO criteria [1] as follows: patient with bone marrow pathology consistent with ET were classified as ET, patients with bone marrow pathology consistent with PMF and at least one clinical criteria (anemia, leukocytosis >= 11 x10^9^/L, palpable splenomegaly, increased LDH) were classified as prePMF, patients with bone marrow pathology consistent with PMF but without clinical criteria were classified as myeloproliferative neoplasms unclassifiable (MPNu).

Evolution to post-ET myelofibrosis was diagnosed according to the criteria of the International Working Group of Myelofibrosis Research and Treatment (IWG-MRT) [26], while evolution into acute myeloid leukemia was defined according to the WHO criteria [10]. Thrombotic events and bleeding were defined as described in detail elsewhere [5, 27].

### *JAK2*, *MPL* and *CALR* mutation analysis

The mutational profile was studied on granulocytes’ DNA from peripheral blood. *JAK2* (V617F) mutation status was assessed using a quantitative polymerase chain reaction (qPCR)–based allelic discrimination assay on a Rotor-Gene 6000 real-time analyzer (Qiagen), as previously described [28].

*JAK2* wild type patients were further evaluated for *CALR* exon 9 mutations and *MPL* exon 10 mutations, as previously reported [29–32].

### Statistical analysis

Numerical variables have been summarized by their median and range, and categorical variables by count and relative frequency (%) of each category. Comparisons of quantitative variables between two or more groups of patients were carried out by the non-parametric Wilcoxon rank-sum test or the Kruskall-Wallis test, respectively. Association between categorical variables (two-way tables) was tested by the Fisher's exact test.

Overall survival (OS) from diagnosis was estimated using the Kaplan-Meier product limit method and survival curves were compared by the log-rank test. The cumulative incidence of myelofibrotic and leukemic transformation, and that of thrombotic complications and bleedings were estimated with a competing risk approach, considering death for any cause as a competing event. The comparison between curves of cumulative incidence was carried-out by Pepe&Mori test or Fine&Gray model (for multivariate models). For the comparison of the hematological characteristics at diagnosis among ET, MPNu, prePMF we considered statistically significant *P* values <.017 (correction for multiple testing considering 3 categories to be compared). The 26 patients with MPNu were not further considered in the analysis of disease complications and overall survival due to the low number. So correction for multiple testing was not necessary in the analysis of outcome and all P-values were considered statistically significant when smaller than 0.05. Statistical analyses were performed using Stata 12.1 (StataCorp LP, College Station, TX) software.
